# The role of ipsilateral motor network in upper limb movement

**DOI:** 10.3389/fphys.2023.1199338

**Published:** 2023-07-03

**Authors:** Hao Ding, Nelly Seusing, Bahman Nasseroleslami, Abdul Rauf Anwar, Sebastian Strauss, Martin Lotze, Matthias Grothe, Sergiu Groppa, Muthuraman Muthuraman

**Affiliations:** ^1^ Department of Neurology, University Hospital Würzburg, Würzburg, Germany; ^2^ Academic Unit of Neurology, Trinity College Dublin, The University of Dublin, Dublin, Ireland; ^3^ Department of Neurology, University Medicine of Greifswald, Greifswald, Germany; ^4^ Paris Brain Institute, Paris, France; ^5^ Functional Imaging Unit, Center for Diagnostic Radiology, University Medicine Greifswald, Greifswald, Germany; ^6^ Department of Neurology, University Medical Center of the Johannes Gutenberg University Mainz, Mainz, Germany

**Keywords:** ipsilateral motor network, upper limb, ipsilateral motor evoked potential, voluntary movement, bilateral motor network

## Abstract

The execution of voluntary movements is primarily governed by the cerebral hemisphere contralateral to the moving limb. Previous research indicates that the ipsilateral motor network, comprising the primary motor cortex (M1), supplementary motor area (SMA), and premotor cortex (PM), plays a crucial role in the planning and execution of limb movements. However, the precise functions of this network and its interplay in different task contexts have yet to be fully understood. Twenty healthy right-handed participants (10 females, mean age 26.1 ± 4.6 years) underwent functional MRI scans while performing biceps brachii representations such as bilateral, unilateral flexion, and bilateral flexion-extension. Ipsilateral motor evoked potentials (iMEPs) were obtained from the identical set of participants in a prior study using transcranial magnetic stimulation (TMS) targeting M1 while employing the same motor tasks. The voxel time series was extracted based on the region of interest (M1, SMA, ventral PM and dorsal PM). Directed functinal connectivity was derived from the extracted time series using time-resolved partial directed coherence. We found increased connectivity from left-PMv to both sides M1, as well as right-PMv to both sides SMA, in unilateral flexion compared to bilateral flexion. Connectivity from left M1 to left-PMv, and left-SMA to right-PMd, also increased in both unilateral flexion and bilateral flexion-extension compared to bilateral flexion. However, connectivity between PMv and right-M1 to left-PMd decreased during bilateral flexion-extension compared to unilateral flexion. Additionally, during bilateral flexion-extension, the connectivity from right-M1 to right-SMA had a negative relationship with the area ratio of iMEP in the dominant side. Our results provide corroborating evidence for prior research suggesting that the ipsilateral motor network is implicated in the voluntary movements and underscores its involvement in cognitive processes such as movement planning and coordination. Moreover, ipsilateral connectivity from M1 to SMA on the dominant side can modulate the degree of ipsilateral M1 activation during bilateral antagonistic contraction.

## 1 Introduction

A long-standing hypothesis that the voluntary limb movement is dominated by the contralateral hemisphere of the moving limb. It finds support in study demonstrating that electrical stimulation of the motor cortex leads to limb movement on the side opposite to the site of stimulation (Fleming, 1938). Furthermore, unilateral damage to the motor cortex often results in motor deficits on the contralateral side of the body (Duncan et al., 2018). Conversely, during unimanual limb movements, several studies have documented activation of motor areas in the same side or ipsilateral hemisphere, including the primary motor (M1) and premotor areas (PM) as opposed to the observed contralateral neural activity (Bundy and Leuthardt, 2019).

Functional imaging investigations have demonstrated the activation of the motor cortex in the same hemisphere as the moving limb. In contrast to the activation observed in the contralateral M1 during unilateral limb movements, the ipsilateral M1 is also active but with a smaller area of activation (Grafton et al., 1992; Kim et al., 1993). The strength of the ipsilateral motor activity has been found to associate with task complexity and the demands for accuracy (Rao et al., 1993; Buetefisch et al., 2014). In the context of unilateral isometric contraction from the dominant hand, there was documented evidence of a discernible bilateral network (Mayhew et al., 2017). Despite the evidence demonstrating the involvement of ipsilateral motor cortex during limb movement, only a limited number of studies have dived deeper into the brain network within the motor cortex. Most commonly, dynamic causal modelling (DCM), which enables the estimation of brain connectivity within a hypothesis-driven framework, is frequently employed to examine the relationships between regions of interest (ROIs). Grefkes and colleagues observed reduced connectivity towards ipsilateral motor areas during unimanual movements and increased connectivity towards the contralateral M1, with highlighted brain region in supplementary area (SMA) (Grefkes et al., 2008). In another study (Volz et al., 2015), the inhibitory effects on the ipsilateral M1 were found strongly influenced by PM and contralateral M1 homologue. However, connectivity analysis based on data-driven approach, which offers valuable alternative by leveraging observed data to infer the connectivity pattern and dynamics of the brain network, is sparsely applied to neuroimaging modalities to investigate the human motor network.

Furthermore, the recruitment of ipsilateral motor pathways can be examined neurophysiologically with transcranial magnetic stimulation (TMS) (Armand and Kuypers, 1980; Wassermann et al., 1994; Ziemann et al., 1999). In contrast to contralateral motor evoked potential (cMEP), ipsilateral motor evoked potential (iMEP) exhibits distinctive characteristics, including a delayed onset, higher activation threshold, and reduced amplitude. These features suggest a weaker and potentially indirect pathway to spinal alpha motor neurons. Notably, iMEPs are more readily elicited in proximal muscles compared to distal muscles (Wassermann et al., 1994; Bawa et al., 2004) and are typically observed when the target muscle is pre-activated (Chen et al., 2003; Bawa et al., 2004). These characteristics underscore the potential significance of iMEPs in the context of bimanual or postural motor interactions. The excitability of iMEPs depending on the task context was previously suggested by Tazoe and colleagues (Tazoe and Perez, 2014). In their study, they identified the largest iMEPs in bilateral contraction of heteronymous muscles, which involved the contraction of the non-dominant arm’s biceps brachii (elbow flexion) concurrently with the contraction of the dominant arm’s triceps brachii (elbow extension). Additionally, iMEP obtained during bilateral elbow flexion from the non-dominant side were smaller compared to those observed during unilateral elbow flexion (Perez et al., 2013; Seusing et al., 2023). However, in a similar paradigm to our recent work (Seusing et al., 2023), we found that the size of the iMEP was not influenced by the different upper limb movements. Hence, the functional significance of iMEP and its precise underlying neural mechanism continue to be unclear and subject to ongoing debate.

Understanding the role of ipsilateral motor activity is crucial for improving our knowledge of how the brain facilitates voluntary movements in healthy individuals and for understanding the potential role played by the contralesional hemisphere during the recovery of ipsilateral motor function after brain injuries such as stroke or traumatic brain injury (Bundy and Leuthardt, 2019). By integrating iMEP assessments and neuroimaging techniques, we can uncover how the excitability of ipsilateral motor activity, as assessed by iMEP, relates to brain connectivity patterns detected through fMRI. This investigation enables a deeper understanding of the neural mechanisms underlying motor control, interhemispheric interactions, and the role of the ipsilateral motor network. It sheds light on the intricate relationship between neural excitability and functional brain activity.

**FIGURE 1:**
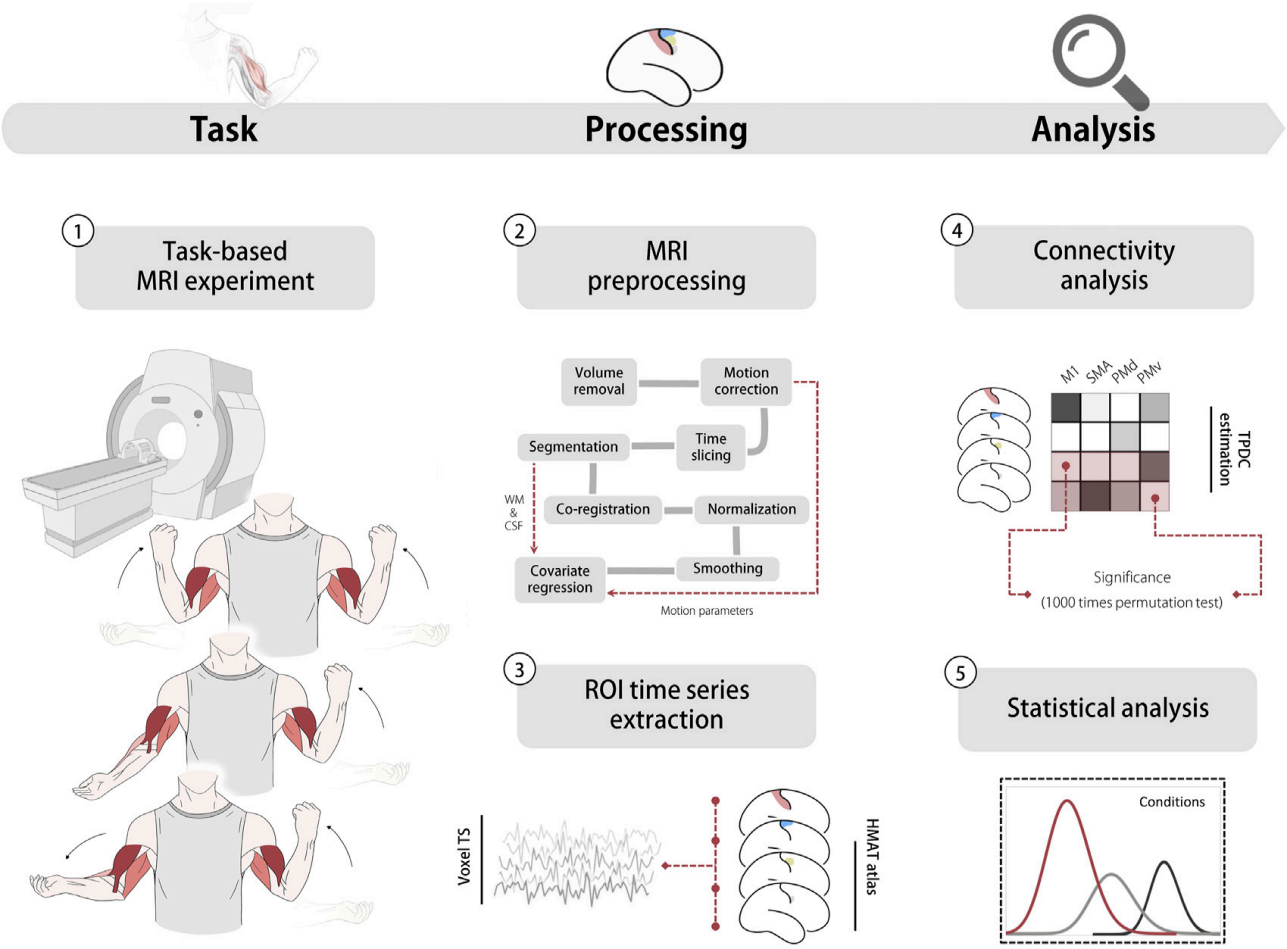
Experiment and analysis pipeline. 1. Task based MRI experiment (top: bilateral flexion; middle: unilateral flexion; bottom: bilateral flexion-extension) 2. MRI pre-processing pipeline, head motion parameters, WM and CSF were used in the covariate regression 3. Time series extracted from ROls. 4 Directed functional connectivity estimation using TPDC based on extracted data. 5 Statistical analysis including ANOVA and correlation analysis. Abbreviation: WM white matter, CSF Cerebrospinal fluid, TS time series, HMAT human motor area template, ROI region of interest, M1 primary motor cortex, SMA supplementary motor area, PMd dorsal premotor, PMv ventral premotor. TPDC time-resolved partial directed coherence.

The present study aimed to build upon our prior research and investigate the neural network interactions within the motor cortex during various task contexts, while also examining their association with iMEP as depicted in Figure 1. We chose three conditions that are comparable to previous studies (Tazoe and Perez, 2014; Seusing et al., 2023) to access whether unilateral, bilateral homologous and bilateral heterologous/antagonistic contexts would reveal differential modulation based on the ROIs (M1, PMv, PMd, SMA) defined by Human motor area template (HMAT). We estimated directed functional connectivity to examine the information flow between these regions using time-resolved partial directed coherence (TPDC) based on fMRI data (Anwar et al., 2013). Finally, we employed a correlation analysis between the directed connectivity and iMEP obtained from TMS (Seusing et al., 2023) to explore the relationship between iMEP and network connectivity. We hypothesis that the connectivity patterns within the motor cortex would reflect directed neural modulations under different task contexts. Furthermore, we anticipate that the involvement of ipsilateral motor areas in network modulation would be more pronounced during complex motor tasks and demonstrate association with iMEP specifically within the complex task contexts.

## 2 Materials and methods

### 2.1 Participant

Twenty healthy volunteers (26.1 ± 4.6 years old, 10 female) enrolled in the present study. All participants were right-handed according to Edinburgh Handedness inventory (mean score 94.3) (Oldfield 1971). None of the participants had any neurological disorder or took any medication. All participants gave written informed consent to the experimental procedures, which were approved by the local ethics committee at the University Medicine of Greifswald (BB139/18).

### 2.2 Experiment procedure

The experiments were implemented as blocked and event-related designs during the MRI scanning. The blocked design was 12 min, consisting of interleaving 12 task blocks and 11 rest blocks with a 30 s duration (Figure 1). The participants performed three different motor tasks: 1) bilateral flexion (homologous contraction): bilateral contraction of both biceps brachii; 2) unilateral flexion: contraction of left biceps brachii and relaxation of the right arm; 3) bilateral flexion-extension (antagonistic contraction): contraction of the left biceps brachii and extension of the right arm. Participants were instructed to perform an isometric contraction or extension at 50% of their maximal voluntary movement and keep their heads straight during the experiment. Muscle force during the MRI was monitored visually and with a dynamometer. Real-time visual feedback was provided to the participants on a screen display to allow them to observe their muscle activity. In addition, a dynamometer was used to measure and quantify the actual force exerted by the muscles during the fMRI sessions. This dual approach provided a comprehensive means of monitoring and controlling the muscle force throughout the study.

### 2.3 iMEP acquisition

The present study included participants who had participated in our prior investigation, which involved a TMS employing identical motor tasks as described in the perimental procedure section, with the aim of obtaining iMEP. During the TMS experiment, participants were seated comfortably in a chair and connected to an electromyography (EMG) system, while also undergoing frameless neuronavigation for registration purposes. The EMG activity was recorded from the left and right biceps brachii muscles using surface electrodes arranged in a tendon-belly-montage configuration, utilizing 10 mm Ag/AgCl electrodes. The recorded EMG signals were subsequently amplified (CED 1902; Cambridge Electronic Design, United Kingdom), band-pass filtered within the range of 20–1,000 Hz, and sampled at a rate of 2 kHz (CED 1401). The data were stored for offline analysis using Signal software (V6.0, CED). The figure-eight coil was applied tangentially to the scalp, oriented at a 45° angle to induce current flow in a posterior to anterior direction. TMS was delivered starting from the anatomical hand knob landmark. Throughout the experiment, participants were instructed to maintain a straight head position at all times and were encouraged to perform an isometric contraction at 50% of their maximum voluntary contraction level in the biceps brachii muscle on the side ipsilateral to the stimulation. The muscle force exerted was visually monitored using a dynamometer, and participants were granted sufficient rest periods within the tasks as needed. For a more comprehensive description of the TMS experiment and setup, we direct the reader to consult the original publication (Seusing et al., 2023).

### 2.4 MRI acquisition

Whole-brain imaging data from all subjects were collected on a 3T scanner (Verio, Siemens, Erlangen, Germany) with a 32-channel head coil. T1-weighted images were acquired using three-dimensional magnetization-prepared rapid gradient-echo imaging with following parameters: repetition time (TR) = 1.69 m; echo time (TE) = 2.52 m; field of view (FoV) = 256 × 256 mm; number of slices = 176, voxel size = 1 × 1 × 1 mm^3^; fMRI data consisted of 360 volumes using single-shot echo-planar imaging (EPI) sequence with TR = 2000 m; TE = 30 m ; FA = 90°; FoV = 256 mm ;voxel size = 3 × 3 × 3 mm^3^.

### 2.5 MRI pre-processing

fMRI data pre-processing steps were performed following Statistical Parametric Mapping (SPM12) guidelines (Esteban et al., 2019; Diaz et al., 2021). For each subject, the first five volumes were removed to account for T1 relaxation effects. Realignment was performed to remove head motion using rigid body translation and rotation followed by slice timing correction and co-registration with T1 image. Scans were then spatially normalized to Montreal neurological institute (MNI) space using the deformation matrices obtained during MRI pre-processing using the CAT12 toolbox (Structural Brain Mapping Group, Jena University Hospital, Jena, Germany) (Gaser and Dahnke, 2016). The scans were smoothed by convolving with Gaussian kernel of fixed width (6 mm full-width half maximum kernel) to suppress noise. In the end, the nuisance covariate regression was performed including six motion correction parameters and averaged white matter (WM) and cerebrospinal fluid signals (CSF). The fMRI time series data underwent visual inspection by experienced neuroscientists to ensure its quality for subsequent analysis. It worth to mention that we did not employ a data-driven artifacts removal method, such as ICA-AROMA (Pruim et al., 2015) in our preprocessing pipeline. This decision was based on our experimental design, which incorporated short task performance runs, thereby reducing the impact of motion artifacts. Consequently, this approach can avoid the aggressive removal of meaningful signal relevant to our task from the independent components. Thus, we considered the inclusion of motion parameters regression, including WM and CSF, as adequate for this study. This regression approach has been widely utilized in fMRI connectivity studies in previous research (Blasi et al., 2020; Fleischer et al., 2020; Diaz et al., 2021; Ding et al., 2022) and was found to better capture temporal degrees of freedom compared to other advanced artifacts removal methods (Blasi et al., 2020). Finally, the time series extraction based on HMAT (Archer et al., 2018) atlas was carried out. Based on previous findings in cortical physiology of limb movements (Bundy and Leuthardt, 2019), our analysis involves both hemispheres and focuses on cortical areas, namely, M1, PMv, PMd, SMA.

### 2.6 Time solved partial directed coherence

In the present study, we used time-resolved partial directed coherence (TPDC) method to estimate directed functional connectivity estimation. This method enables us to focus on the temporal dynamics of a signal and analyze directional influence at any specific frequency band. It relies on dual-extended Kalman filtering (DEKF) (Wan and Nelson, 2001), which is one of the most widely used estimation algorithms for nonlinear systems, to calculate time-dependent multivariate autoregressive (MVAR) coefficients at each time point. Briefly, one extended Kalman filter (EKF) estimates the states and feeds this information to the other. The second EKF estimates the model parameters and shares this information with the first. By using two Kalman filters working in parallel, we can estimate both the states and model parameters of the system at each time instant. For detailed DEKF algorithm, we refer reader to the original publication (Wan and Nelson, 2001). Subsequently, Fourier transformation was applied to the MVAR coefficients and computed partial directed coherence (PDC) for each time point. PDC from time-series *x*_*j*_ to *x*_*i*_ at each time point can be calculated by:

$$\pi_{i\leftarrow j}\left(f\right)=\frac{\left|A_{ij}\left(f\right)\right|}{\sqrt{\sum_{k=1}^{N}{\left|A_{kj}\left(f\right)\right|}^{2}}}$$

where *A*_*ij*_ refers to the Fourier transformed MVAR coefficients matrix and *N* refers to the number of the connections. In the fMRI time series, we extracted the frequency band of interests from 0.009 to 0.08 Hz and averaged across each time point to obtain robust connectivity values between different motor areas. The choice of this frequency range is based on several factors. Firstly, it is known that neuronal activity in the brain exhibits low-frequency oscillations (<0.1 Hz) and believed to reflect functional networks that are active in that region during task performance (Biswal et al., 1995; Lowe et al., 1998; Cordes et al., 2000; Raichle et al., 2001; Greicius et al., 2004; Roy et al., 2009; Biswal et al., 2010). Secondly, the BOLD signal is relatively slow, with changes occurring over several seconds, which is believed to be related to underlying neuronal activity and functional connectivity in the brain (Fox and Raichle, 2007). The low-frequency range (0.009–0.08 Hz) has been used in numerous fMRI studies to investigate functional connectivity in the human brain (Biswal et al., 1995; Fox et al., 2005; Fox and Raichle, 2007; Ding et al., 2022). Since the precise distribution of the MVAR coefficients is not known, we used a surrogate approach to check the significance of the results. In short, we randomly shuffled the order of these task blocks to create a new time series. The TPDC value is calculated based on a randomly shuffled time series for 1,000 times and the 95th percentile of the connectivity value was taken as the significance threshold. This process is performed separately for each subject.

### 2.7 Statistical analysis

Statistical analysis of the connectivity data was performed in RStudio (R version 4.1.2). Each connectivity was compared between conditions using non-parametric repeated measure ANOVA. The level of statistical significance against the null-hypothesis was set to *p* < 0.05 (two-tailed). Wilcoxon test was performed as *post hoc* method with Bonferroni correction.

### 2.8 Correlation analysis

In order to examine the correlation between the task-related directed functional connectivity and the iMEP parameters, Spearman’s correlation (Cohen et al., 2009) was performed on the directed functional connectivity and the iMEP measurements (iMEP duration, amplitude, and area) obtained from our recent publication. The level of statistical significance against the null hypothesis was set to *p* < 0.01. It is worth mentioning that the iMEP area ratio was calculated using the following formula: [area of rectified electromyography (EMG) in iMEP duration/(mean prestimulus EMG*iMEP duration) *100] (Tazoe and Perez, 2014), thus iMEP area ratio expressing the relative size of iMEP compared to the prestimulus EMG. For more detailed iMEP data processing, we refer the reader to the original publication (Seusing et al., 2023).

## 3 Results

### 3.1 Ipsilateral motor evoked potential parameters

As shown in Table 1, unilateral flexion shows the highest mean iMEP duration, iMEP area and area ratio. The highest iMEP amplitude was observed during bilateral flexion-extension task.

**TABLE 1:** iMEP parameters under different motor tasks. Abbreviation: IMEP (ipsilateral motor evoked potential), ms millisecond, μV μV, ‰ permille, % percentage.

IMEP parameters under different motor task		Mean (±standard error)
Duration (ms)		
	Bilateral flexion	19.6 (4.7)
	Unilateral flexion	26.2 (5.1)
	Bilateral flexion-extension	20 (3.5)
Amplitude (μV)		
	Bilateral flexion	285.9 (63.5)
	Unilateral flexion	266.5 (46.1)
	Bilateral flexion-extension	303 (48.1)
Area (‰)		
	Bilateral flexion	7.1 (3.2)
	Unilateral flexion	8.6 (4.5)
	Bilateral flexion-extension	5.6 (1.4)
Area ratio (%)		
	Bilateral flexion	219.8 (32.8)
	Unilateral flexion	230.1 (42.6)
	Bilateral flexion-extension	196.1 (14.2)

### 3.2 Difference of neural modulation in unilateral flexion, bilateral flexion-extension, in comparison with bilateral flexion

**FIGURE 2:**
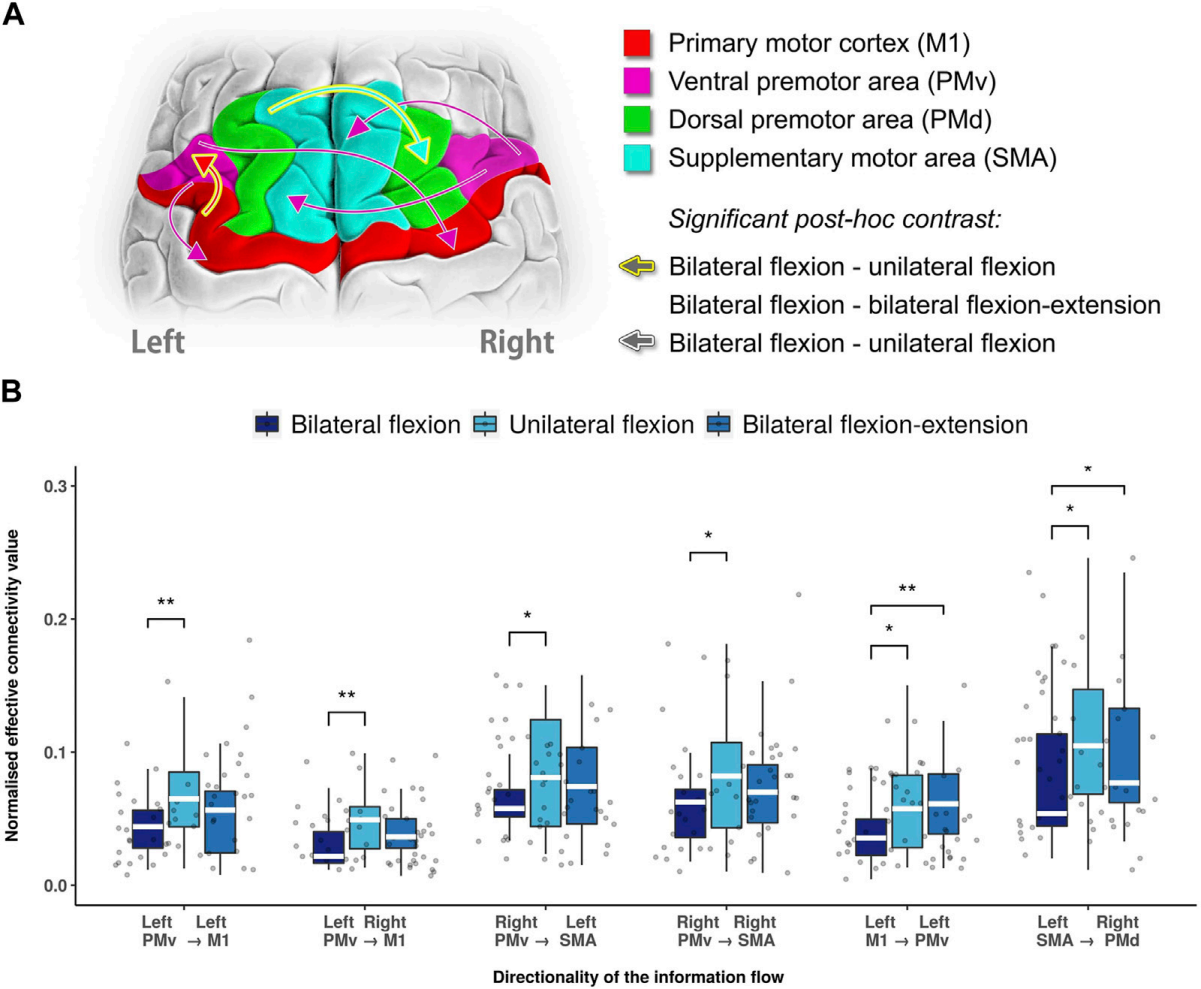
Statistical significance summary of directed functional connectivity between different motor tasks. **(A)** Significant directed functional connectivity between different motor tasks revealed by Wilcoxon test (Bonferroni corrected). In this illustration, the arrows stroked with white colour indicates the distinct directed functional connectivity between unilateral and bilateral flexion, while a golden stroked arrow indicates both bilateral flexion-extension and unilateral flexion as distinct from bilateral flexion. **(B)** Boxplot of significant directed functional connectivity summary. Abbreviations: Ml: primary motor cortex; PMv: ventral premotor area; PMd: dorsal premotor area; SMA: supplementary motor area.; white line (within the box): median level. (*): *p* < 0.05; (**): *p* < 0.01.

Within selected motor areas, non-parametric repeated ANOVA revealed six directed functional connectivity that were statistically significant between three different motor tasks as shown in Figure 2. In the unilateral flexion task, where the left arm performed elbow flexion and the right arm was at rest, we observed a significant increase in information flow from the left PMv to both sides M1 (left M1: *p* = 0.0014; right M1: *p* = 0.0043, Bonferroni corrected) compared to bilateral contraction. Likewise, the information flow from right PMv to both left SMA (*p* = 0.0408, Bonferroni corrected) and right SMA (*p* = 0.3624, Bonferroni corrected) were found increased as well. Moreover, ipsilateral connectivity from left M1 to left PMv (*p* = 0.0362, Bonferroni corrected) and inter-hemispheric connectivity from left SMA to right PMd (*p* = 0.0110, Bonferroni corrected) were also increased compared to bilateral flexion. Furthermore, these two ECs were also found to be significantly higher in bilateral flexion-extension task compared with bilateral flexion: left M1 to left PMv (*p* = 0.0095, Bonferroni corrected) and left SMA to right PMd (*p* = 0.0249, Bonferroni corrected) as shown in Figure 2B. The detailed results are summarized in the Supplementary Table S1.

### 3.3 Difference between unilateral flexion and bilateral flexion-extension

**FIGURE 3:**
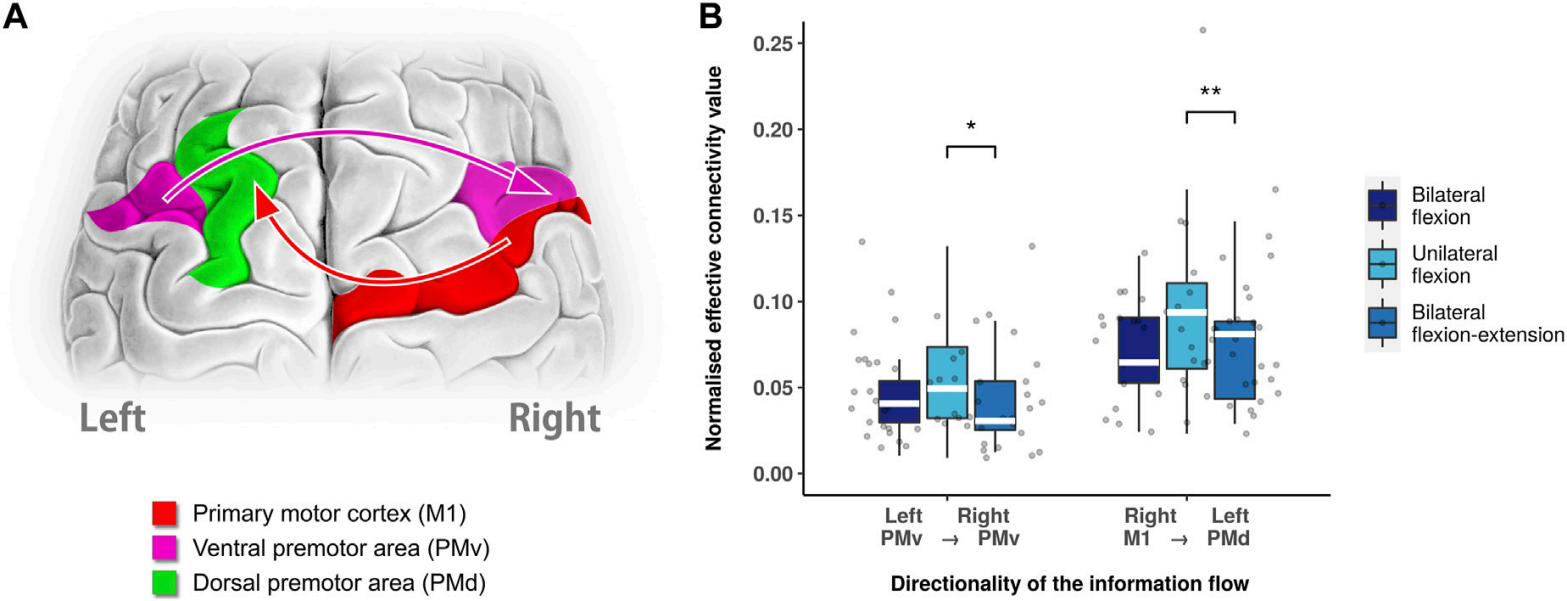
Different neural modulation between unilateral flexion and bilateral flexion-extension. **(A)** Significant directed functional connectivity between unilateral flexion and bilateral flexion-extension task revealed by Wilcoxon test (Bonferroni corrected). **(B)** Boxplot of significant directed functional connectivity summary. Abbreviations: Ml: primary motor cortex; PMv: ventral premotor area; PMd: dorsal premotor area; SMA: supplementary motor area.; white line (within the box): median level. (*): *p* < 0.05; (**): *p* < 0.01.

To further reveal the difference in connectivity between unilateral flexion and bilateral flexion-extension, *post hoc* analysis with Wilcoxon test revealed a decreased level of inter-hemispheric connectivity from left PMv to right PMv (*p* = 0.0283, Bonferroni corrected) and right M1 to left PMd (*p* = 0.0304, Bonferroni corrected) during bilateral flexion-extension compared with unilateral flexion as shown in Figure 3.

### 3.4 Correlation between directed functional connectivity and iMEP parameters

**FIGURE 4:**
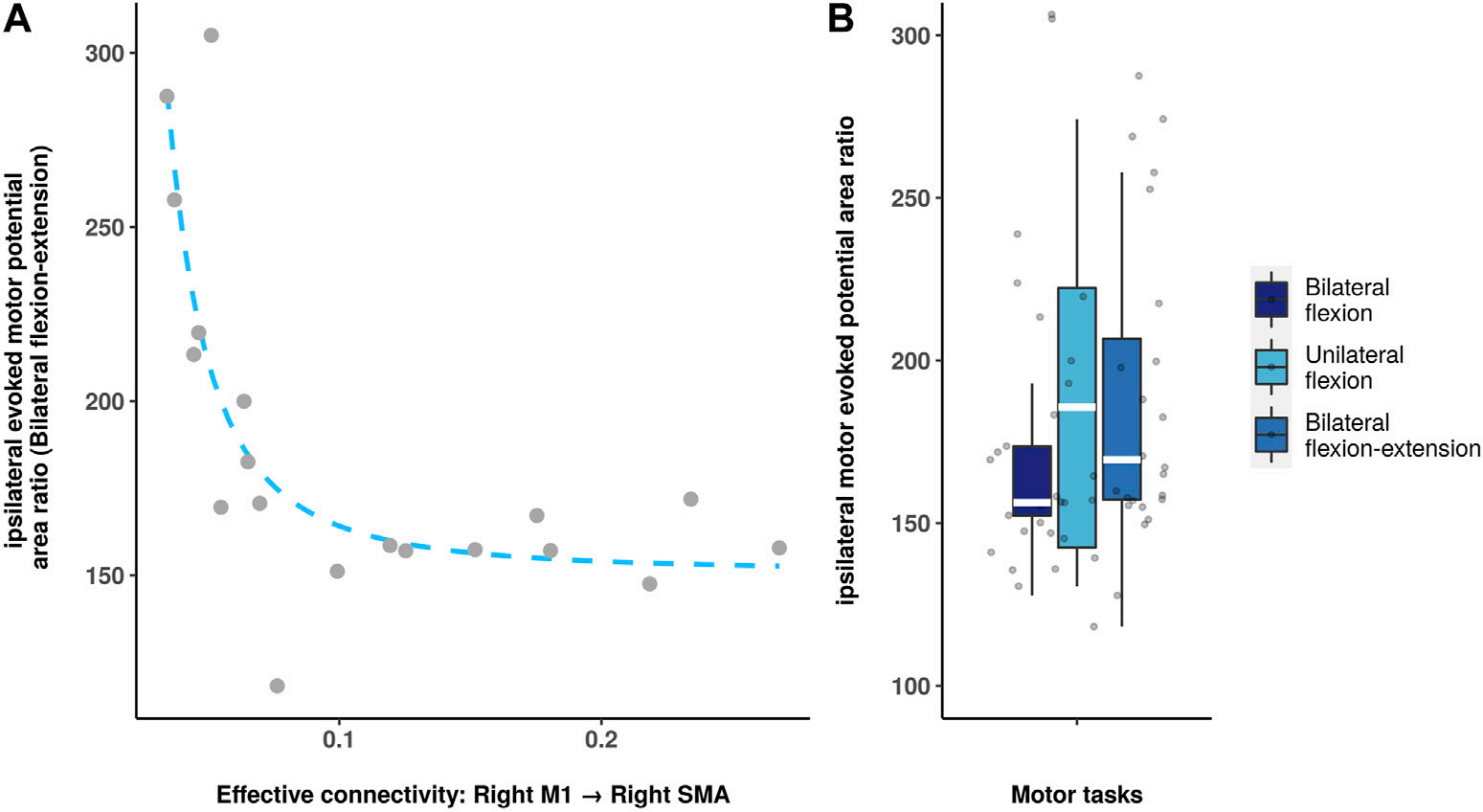
Spearman correlation analysis between directed functional connectivity and iMEP parameters. **(A)**: Scatter plot shows the significant correlation between iMEP area ratio and the connectivity (right M1 to the right SMA). **(B)**: Boxplot shows the iMEP area ratio during different motor tasks.

Figure 4 shows a negative correlation between directed functional connectivity in right M1 to right SMA and iMEP area ratio (*p* = 0.0094, Bonferroni correction) during bilateral flexion-extension task. No statistical significance was observed during bilateral and unilateral flexion task. The rest correlation results are summarized in the (Supplementary Figure S1).

## 4 Discussion

Our study contributes to expanding our understanding of the modulatory mechanism involved in voluntary movements. By demonstrating the involvement of ipsilateral motor network and highlighting functional connectivity related MEP. We provide novel insights into the active and regulatory role of this network across different motor tasks. Consequently, our findings enhance our understanding of the intricate dynamics that govern voluntary movements.

The neural modulations within the motor cortex during different motor tasks are not yet fully understood. Precisely quantifying directed functional connectivity, which is based on interactions between relevant brain regions within the motor cortex, can provide a deeper understanding of the function of the ipsilateral hemisphere in voluntary movements. Previous research suggested that the iMEP may be task-specific and spatially distinct from the contralateral hotspot (Seusing et al., 2023). However, our study identified distinct connectivity patterns for bilateral, unilateral, and bilateral flexion-extension movements involving both hemispheres. Additionally, a specific directed functional connectivity pattern (right M1 to right SMA) derived from fMRI time series was found to have a negative correlation with the MEP area ratio, offering further insights into the complex motor networks involved.

The premotor cortex has long been studied in primates, yet the functions of the premotor cortex are diverse and not fully understood (Wise, 1985). In primates, PMv projects to the hand and arm fields of M1, which contains the largest and most detailed representation of hand movements among all cortical motor areas. Additionally, its sensory representation has been shown to contribute to the planning and execution of movements (Kandel et al., 2012). Between bilateral and unilateral flexion, we found that not only the contralateral but also the ipsilateral M1 connections desynchronized from PMv as the strength of the information flow decreased. This highlights the engagement of ipsilateral M1 in the representation of biceps brachii contraction. Previous studies reported that the involvement of ipsilateral M1 in hand movement is often associated with more complex tasks (Chen et al., 1997a; Chen et al., 1997b). However, recent studies in both animals and humans have confirmed the conventional view that ipsilateral projections from M1 to the upper limb are directed to truncal and proximal muscles, which are mainly located in the upper arm. The observation of connectivity from the left PMv to both sides of M1 indicates that M1 functions bilaterally while still being driven by the PMv, even during unilateral flexion task. Furthermore, the lower median level of connectivity towards right M1 (left PMv to right M1) compared with left M1 (left PMv to left M1) might be the indicative of laterality (i.e., as in our study in right-handed individuals).

Interestingly, our results highlighted another bi-hemispheric relationship from right PMV to both sides of SMA as the level of connectivity was significantly decreased during the bilateral flexion task compared with unilateral flexion. In SMA, neurons often relate to complex actions such as postural adjustments and movements that require switching between actions, plans and strategies (Kandel et al., 2012). The projection from PMv to SMA was recently reported during grasping movements. This study proposed a role for integration for force variation at the high-level processing stage (Haller et al., 2009). Due to its direct projections to the hand and motor neurons (Dum and Strick, 1996; Maier et al., 2002), SMA can influence the motor programming without being transmitted to M1 and PMv (Bencivenga et al., 2021). In this study, we observed a decreased connectivity (right PMv to bilateral SMA) during bilateral flexion compared to unilateral flexion, which indicates less modulation recruited from right PMv, suggesting an independent functioning during biceps brachii contraction. Given the role of SMA mainly involved in sensory processing, cognitive and fine motor tasks, the right PMv seem to drive the continuous adjustment and coordination of the voluntary movements.

In addition, we identified two connectivity (left M1 to left PMv; left SMA to right PMd as shown in Figure 2) that distinguish the right arm at flexion, rest and extension, while left arm is in flexion all the time. The projections between PMv and M1 in motor representation was documented in previous studies (Dum and Strick, 2005; Sel et al., 2021). M1 and PM including both dorsal and ventral areas, were previously thought to form a densely interconnected network of cortical areas involved in the generation and control of hand movements. In a Go and no-go motor task, both projections from PMv to M1 and from M1 to PMv were observed, indicating a neural modulation in the reciprocal direction. Likewise, our results demonstrate a higher information flow from M1 to PMv during unilateral flexion and bilateral flexion-extension tasks compared with bilateral flexion, suggesting not only the proxy role of PMv driving M1 as discussed above, but might also play a role in voluntary movements that are dependent on motor context as well.

Furthermore, another inter-hemispheric connection, from left SMA to right PMd, varied in response to changes in the task context. This shows the importance and sensitivity of the PMd in directed functional connectivity analysis, as it was influenced by SMA. In early studies, single-pulse TMS interfered with ipsilateral PMd in patients with acquired lesions, showing an association with the degree of motor impairment and involvement of complex performance (Johansen-Berg et al., 2002; Lotze et al., 2006; Groppa et al., 2012). This association might indicate a compensatory role of ipsilateral PMd in patients with a movement disorder (Stoeckel and Binkofski, 2010). These findings underscore not only the involvement of ipsilateral PMd in the complex task but also a secondary role in the movement when there is dysfunction in the motor network. In addition, one study in finger movement suggests a relationship between SMA and right PMd to relate to bimanual coordination. A recent study (Verstraelen et al., 2021) assessed the planning and executing of bimanual movements via rTMS indicating left PMd increases movement speed and improves movement accuracy whereas right PMd induced deterioration of movement stability. Their results suggest right PMd fulfils a role in continuous adjustment processes of movement. Thus, the involvement of inter-hemispheric connectivity between SMA and PMd is likely to be maintaining an ongoing movement which requires continuous adjustment.

A number of previous studies have documented a reciprocal and interhemispheric relationship between the left and the right PMv (Rushworth et al., 2003; Fujiyama et al., 2016; Verstraelen et al., 2021). As compared to unilateral and bilateral flexion, the bilateral flexion-extension task reduced unidirectional connectivity (left PMv to right PMv), suggesting a specific lateralized modulation during the extension context. It is noteworthy that this connectivity did not differ between bilateral and unilateral flexion. Thus, it is likely to be a distinct feature between homologous and antagonistic contraction.

As mentioned in the above section, PMd was shown to possess the potential to compensate for motor dysfunction and involvement in motor planning. However, the function of PMd is lateralized as left PMd is dominant in motor planning regardless of which hand is moved (Schluter et al., 1998; Schluter et al., 2001; Rushworth et al., 2003; Fujiyama et al., 2013; Fujiyama et al., 2016), whereas right PMd is modulated by task complexity (Groppa et al., 2012). Given the association between M1 and PMd and bimanual performance (Fujiyama et al., 2016; Babaeeghazvini et al., 2019), our finding in the reduction of connectivity from right M1 to left PMd during bilateral flexion-extension suggests an extensive adjustment in the ongoing movement compared with bilateral flexion.

The iMEP parameters derived from TMS experiments exhibited a trend that was similar to the directed functional connectivity findings. Specifically, unilateral flexion demonstrated a greatest iMEP area ratio than the other tasks. Moreover, a noteworthy finding was that the ipsilateral directed functional connectivity from right M1 to right SMA was negatively associated with the iMEP area ratio during the bilateral flexion-extension task, which exhibited an iMEP area ratio higher than that in bilateral flexion but lower than that in unilateral flexion. This negative correlation implies that the modulation from M1 to SMA affects the level of ipsilateral M1 activation during the bilateral antagonistic contraction task, and that the iMEP area ratio may rise exponentially at certain levels of directed functional connectivity. It is noteworthy that no significant correlation was observed between directed functional connectivity and MEP in the context of bilateral and unilateral flexion tasks. This lack of association may be explained by the possibility that the bilateral flexion-extension task necessitates greater interhemispheric communication and coordination, resulting in heightened dependence on the ipsilateral hemisphere and increased modulation from M1 to SMA. Moreover, the unique neural mechanisms engaged in the execution of the bilateral flexion-extension task may contribute to the observed correlation. Nonetheless, additional investigation is warranted to gain a more comprehensive understanding of the underlying mechanisms driving this particular relationship.

This study has several limitations that should be considered when interpreting the results. The directed connectivity methods based on Granger causality require high-temporal resolution data. However, the study relies on low-temporal resolution fMRI data, a significant limitation of the presented results. In addition, fMRI relies on the assumption that the BOLD signal is a reliable proxy for neuronal activity. There are several factors can influence the BOLD signal, such as vascular changes, motion artifacts, and individual variations in neurovascular coupling. These factors can introduce noise and confounds into the fMRI data, potentially affecting the interpretation of the results. In this study, we focus on the low-frequency range commonly used in fMRI connectivity studies, which avoids more susceptible noise and physiological artifacts at high-frequency range. Nevertheless, there is growing evidence demonstrating meaningful network patterns at high-frequency range via high-speed fMRI (Lin et al., 2015; Trapp et al., 2018; Cabral et al., 2023). Therefore, exploring brain network organization in the high-frequency range during upper limb movement might offer valuable insights into the underlying neuronal mechanism, providing complementary information to enhance our understanding. As exploring high-frequency range connectivity is beyond the scope of the present study, further research is needed to fully investigate and elucidate the role of high-frequency components in the context of upper limb motor control.

In summary, the findings of our study shed light on the intricate neural mechanisms underlying upper-limb movements and the role of the ipsilateral motor region in voluntary movements. The observed negative correlation between ipsilateral connectivity from M1 to SMA and iMEP area ratio during the bilateral flexion-extension task suggests a potential modulatory effect of this neural pathway on the degree of ipsilateral M1 activation. These results contribute to a deeper understanding of the functional organization of the motor cortex and provide a foundation for future research on the neural mechanisms underlying voluntary movements.

## Data Availability

The original contributions presented in the study are included in the article/Supplementary Material, further inquiries can be directed to the corresponding authors.
