# Supplementary material for: The role of ipsilateral motor network in upper limb movement
Source: Front Physiol. 2023 Jul 3;14:1199338. doi: 10.3389/fphys.2023.1199338 (PMC10351419; doi:10.3389/fphys.2023.1199338)
Supplement: Supplementary file 1 [file DataSheet1.docx]

Supplementary Material

The role of ipsilateral motor network in upper limb movement

# Supplementary Table

**Table S1.** Non-parametric repeated ANOVA with post-hoc summary

| Connectivity | ANOVA  p-value | Post-hoc | | |
| --- | --- | --- | --- | --- |
|  |  | Bilateral flexion vs.  unilateral flexion | Bilateral flexion  vs. bilateral flexion-  extension | Unilateral flexion  vs. bilateral flexion-  extension |
| Left M1 → Left PMv | **0.008825672** | **0.036237717** | **0.009458542** | 1 |
| Left M1 → Right PMd | **0.019063396** | 0.1453228 | 0.191167831 | 1 |
| Left PMd → Right PMd | **0.014498802** | 0.098304749 | 0.64850235 | 1 |
| Left PMv → Left M1 | **0.000875483** | **0.001447678** | 0.291921616 | 0.07185173 |
| Left PMv → Left SMA | **0.022093067** | 0.247650146 | 0.1453228 | 1 |
| Left PMv → Right M1 | **0.005345098** | **0.004297256** | 0.132162094 | 1 |
| Left PMv → Right PMv | **0.007043979** | 0.191167831 | 0.369277954 | **0.028306961** |
| Left PMv → Right SMA | **0.011348902** | 0.098304749 | 0.119968414 | 0.64850235 |
| Left SMA → Right PMd | **0.003969517** | **0.01096344** | **0.024925232** | 1 |
| Right M1 → Left PMd | **0.010432858** | 0.079925537 | 1 | **0.00304985** |
| Right M1 → Right SMA | **0.025927325** | 0.191167831 | 0.088726044 | 1 |
| Right PMv → Left M1 | **0.03329856** | 0.935382843 | 0.64850235 | 0.079925537 |
| Right PMv → Left SMA | **0.0200295** | **0.040849686** | 0.1453228 | 0.316194534 |
| Right PMv → Right SMA | **0.013352521** | **0.036237717** | 0.369277954 | 0.088726044 |
| Left M1 → Left PMd | 0.260428 | 0.882757187 | 0.64850235 | 1 |
| Left M1 → Left SMA | 0.100188264 | 0.568044662 | 0.291921616 | 1 |
| Left M1 → Right M1 | 0.78725926 | 1 | 1 | 1 |
| Left M1 → Right PMv | 0.199025605 | 0.057701111 | 1 | 0.935382843 |
| Left M1 → Right SMA | 0.544362105 | 1 | 1 | 1 |
| Left PMd → Left M1 | 0.148543428 | 0.208740234 | 1 | 0.132162094 |
| Left PMd → Left PMv | 0.817892581 | 0.989948273 | 1 | 1 |
| Left PMd → Left SMA | 0.087783679 | 0.64850235 | 0.079925537 | 0.460939407 |
| Left PMd → Right M1 | 0.795379812 | 1 | 1 | 1 |
| Left PMd → Right PMv | 0.316645582 | 0.736461639 | 0.935382843 | 1 |
| Left PMd → Right SMA | 0.256897818 | 0.882757187 | 1 | 1 |
| Left PMv → Left PMd | 0.684723711 | 1 | 1 | 1 |
| Left PMv → Right PMd | 0.299715646 | 1 | 1 | 0.428718567 |
| Left SMA → Left M1 | 0.658833806 | 1 | 1 | 1 |
| Left SMA → Left PMd | 0.958824054 | 1 | 1 | 1 |
| Left SMA → Left PMv | 0.442233935 | 1 | 0.832065582 | 1 |
| Left SMA → Right M1 | 0.318245288 | 1 | 1 | 0.882757187 |
| Left SMA → Right PMv | 0.15828781 | 0.108701706 | 1 | 0.341960907 |
| Left SMA → Right SMA | 0.561412044 | 1 | 1 | 0.607349396 |
| Right M1 → Left M1 | 0.434679803 | 0.607349396 | 1 | 0.783296585 |
| Right M1 → Left PMv | 0.083561464 | 0.132162094 | 0.159507751 | 1 |
| Right M1 → Left SMA | 0.061796661 | 0.088726044 | 0.079925537 | 1 |
| Right M1 → Right PMd | 0.368044628 | 1 | 0.691537857 | 1 |
| Right M1 → Right PMv | 0.057421465 | 0.088726044 | 0.341960907 | 0.935382843 |
| Right PMd → Left M1 | 0.911986945 | 1 | 1 | 1 |
| Right PMd → Left PMd | 0.111389045 | 0.64850235 | 0.079925537 | 1 |
| Right PMd → Left PMv | 0.518488186 | 1 | 0.460939407 | 1 |
| Right PMd → Left SMA | 0.391707782 | 1 | 1 | 1 |
| Right PMd → Right M1 | 0.051455536 | 0.316194534 | 0.014579773 | 0.783296585 |
| Right PMd → Right PMv | 0.375579167 | 1 | 0.832065582 | 1 |
| Right PMd → Right SMA | 0.760028887 | 1 | 1 | 1 |
| Right PMv → Left PMd | 0.840176258 | 1 | 0.783296585 | 1 |
| Right PMv → Left PMv | 0.291270435 | 1 | 1 | 0.832065582 |
| Right PMv → Right M1 | 0.414333657 | 0.64850235 | 1 | 1 |
| Right PMv → Right PMd | 0.320247108 | 1 | 0.341960907 | 1 |
| Right SMA → Left M1 | 0.653324966 | 0.64850235 | 1 | 0.783296585 |
| Right SMA → Left PMd | 0.770833445 | 1 | 0.882757187 | 1 |
| Right SMA → Left PMv | 0.917597649 | 1 | 1 | 1 |
| Right SMA → Left SMA | 0.065401816 | 0.53055954 | 0.098304749 | 1 |
| Right SMA → Right M1 | 0.170391803 | 1 | 0.568044662 | 0.26908493 |
| Right SMA → Right PMd | 0.238952539 | 0.227554321 | 0.783296585 | 1 |
| Right SMA → Right PMv | 0.94731235 | 1 | 1 | 0.935382843 |

**Figure S1.** Correlation analysis between connectivity and iMEP parameters


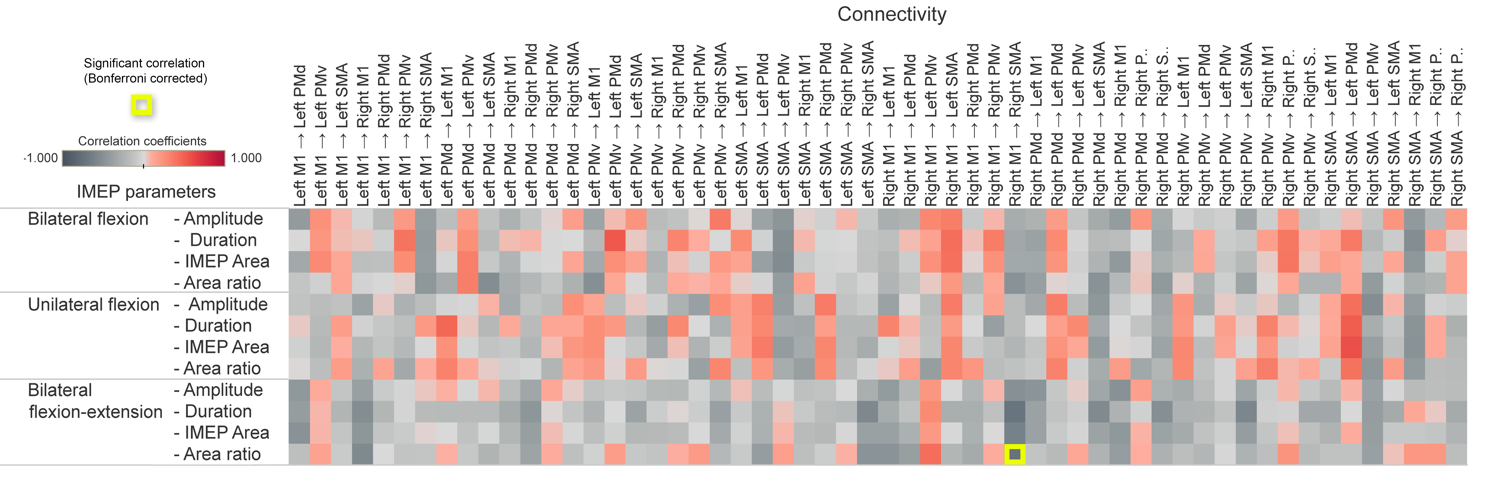


Figure S1 Correlation analysis between connectivity and iMEP parameters. The grid shows the correlation between connectivity (x-axis) and iMEP (y-axis). The correlation coefficient colour coded by the red (positive correlation) and gray (negative correlation). The statistical significance after Bonferroni correction is label with yellow frame.
